# Supplementary material for: A liquid chromatography-mass spectrometry method to quantify total Coenzyme A concentration and isotopic labeling
Source: bioRxiv. 2026 May 20:2026.05.19.726225. Preprint. [Version 1] doi: 10.64898/2026.05.19.726225 (PMC13228548; doi:10.64898/2026.05.19.726225)

Supporting Information

A liquid chromatography-mass spectrometry method to quantify total Coenzyme A concentration and isotopic labeling

Amelia L. Taylor[1], Nathaniel W. Snyder[2], Caroline R. Bartman[1]*

[1]Department of Systems Pharmacology and Translational Therapeutics, Perelman School of Medicine, University of Pennsylvania

[2]Aging and Cardiovascular Discovery Center, Department of Cardiovascular Sciences, Lewis Katz School of Medicine at Temple University

*Corresponding Author Email: cbartman@pennmedicine.upenn.edu

<u>Table of Contents</u>

P2—Table S1: Acyl-CoA standard concentrations

P3—Figure S1: Pre and post hydrolysis and derivatization peak areas for acyl-CoA and CoA-NEM

P4—Figure S2: Hydrolysis and derivatization of short and long chain acyl-CoA

P5—Figure S3: Hydrolysis and derivatization of stable isotope labeled acyl-CoA

P6—Figure S4: Carryover

P7—Figure S5: Blank pseudo-matrix CoA signal

P8—Figure S6: Protein concentration in pseudo-matrix versus tissue matrix

P9—Figure S7: Raw labeling in stable isotopically-labeled pantothenate infusion study

Supplemental Table S1. Absorbance of acyl-CoA standard solutions by UV-Vis absorption spectrophotometry and resulting calculated concentrations.

Standard	Vendor and Catalog Number	Absorbance of 1:200 diluted stock at 259nm	Calculated Stock Concentration
Palmitoyl-CoA	Avanti Polar Lipids 870716P	0.2735	3.8177 mM
Acetyl-CoA	MedChemExpress HY-113596A	0.3869	5.4 mM
Succinyl-CoA	MedChemExpress HY-148285	0.2765	3.859 mM

Supplemental Figure S1. Integrated peak areas before and after hydrolysis and derivatization of acetyl- and succinyl-CoA, corresponding to Figure 2. N=2 independent samples without (blue) and with (red) hydrolysis and NEM derivatization procedure on 50µM acetyl-CoA (left) and 50 µM succinyl-CoA (right) standards.

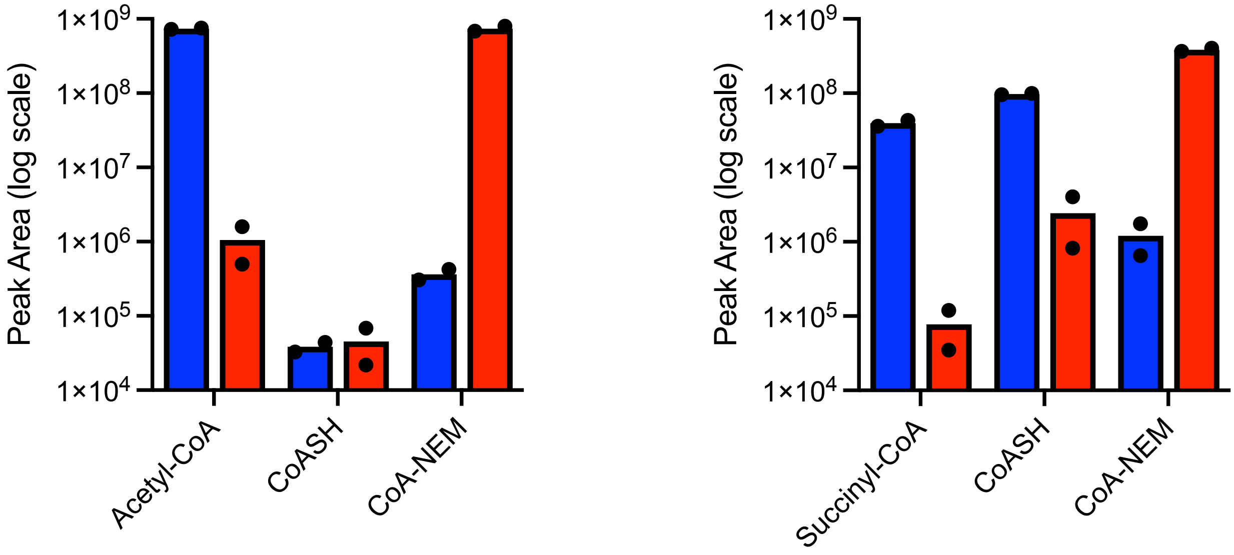

Supplemental Figure S2. Comparative hydrolysis and derivatization between short- and long- chain CoAs. Left) Representative chromatogram for CoA-NEM from hydrolysis and NEM derivatization of 50 μM palmitoyl-CoA standard. **Right)** Peak areas for CoA-NEM from hydrolysis and NEM derivatization procedure carried out on 50 μM standards of acetyl-CoA, succinyl-CoA, or palmitoyl-CoA (n=2 per condition). Data shown as mean and standard deviation.

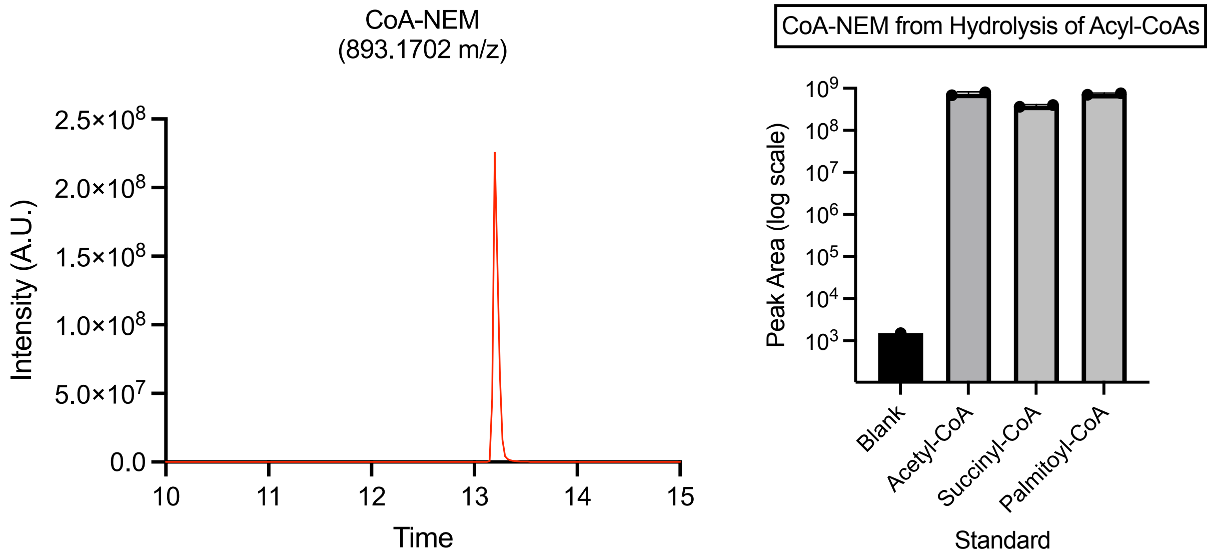

Supplemental Figure S3. Hydrolysis and derivatization of stable isotopically labeled CoA internal standard mix. Representative chromatograms for acetyl-CoA and CoA-NEM without (blue) and with (red) hydrolysis and NEM derivatization procedure, from 50µM acetyl-CoA standards with acyl-CoA heavy isotopically labeled internal standard. Top shows unlabeled standard, and reflection plot shows [$^{13}C_3$-$^{15}N_1$] labeled.

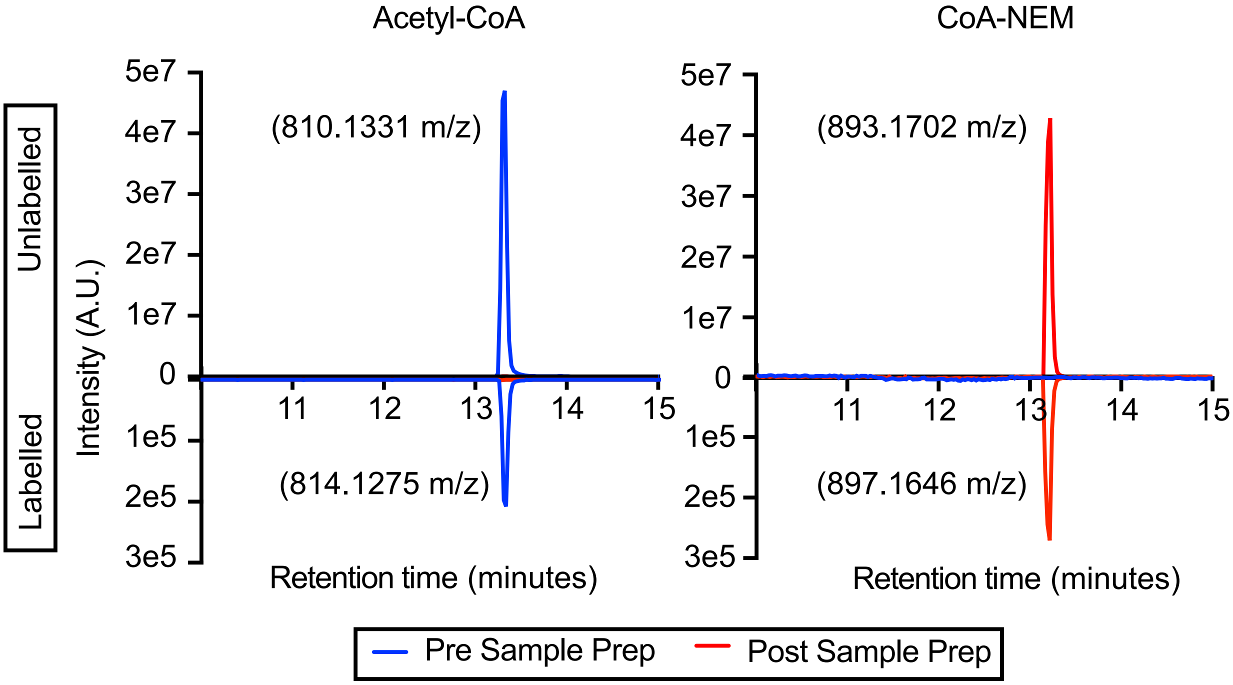

Supplemental Figure S4. Validation of minimal carryover from analysis of hydrolyzed and derivatized acetyl-CoA. Raw peak area of n=3 blanks (injected post highest calibrator) and lowest concentration calibrator. Data shown as mean and standard deviation, plotted on a log scale.

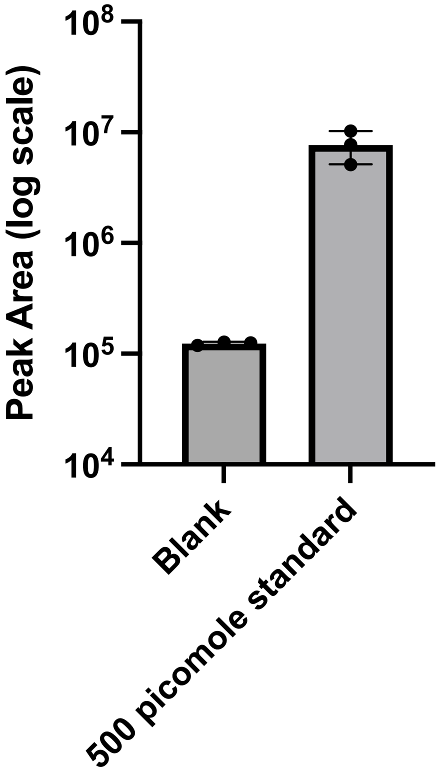

Supplemental Figure S5. CoA-NEM signal from hydrolyzed and derivatized blank serum. Raw peak area of 60% fetal bovine serum with no added CoA standard and lowest concentration calibrator. N=3 per bar, data shown as mean and standard deviation, plotted on a log scale.

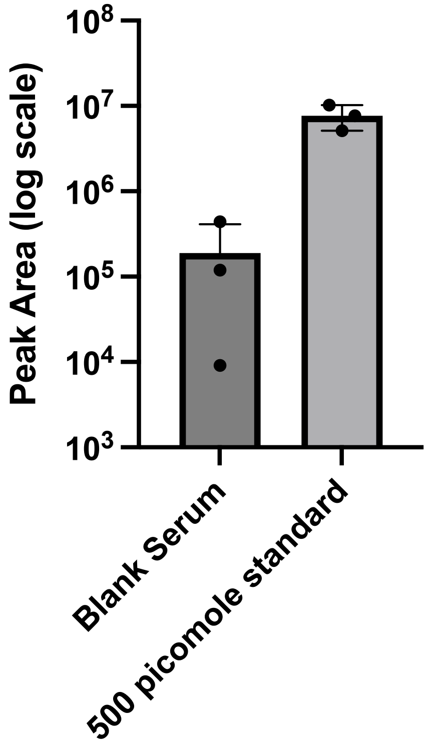

Supplemental Figure S6. Protein concentration in true matrix (tissue) versus pseudo-matrix (serum) used to determine concentration of serum in calibrators and quality control samples. Protein concentration by bicinchoninic acid assay for n=3 aliquots of fetal bovine serum and n=3 mouse liver tissue samples. Data shown as mean and standard deviation.

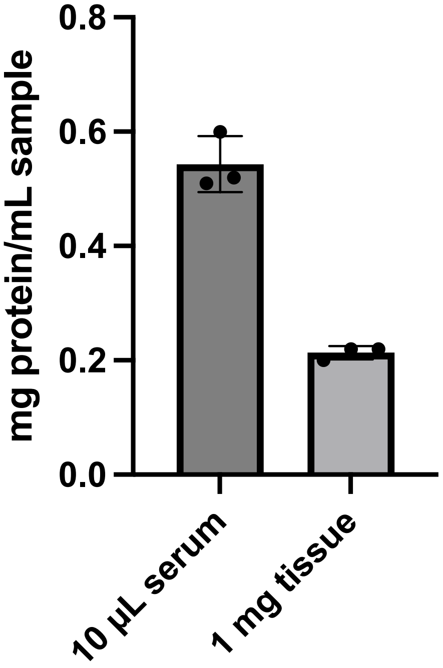

Supplemental Figure S7. Labeling for serum pantothenate and for liver CoA-NEM from pantothenate intravenous infusion, used to calculate serum-normalized liver CoA-NEM labeling shown in Figure 4E. [$^{13}C_3$-$^{15}N_1$] labeled fraction of total for serum pantothenate (black bar) and mouse liver tissue CoA-NEM (gray bar) from mice infused for 24 or 48 hours with [$^{13}C_3$-$^{15}N_1$] labeled pantothenate. n=1 per bar.

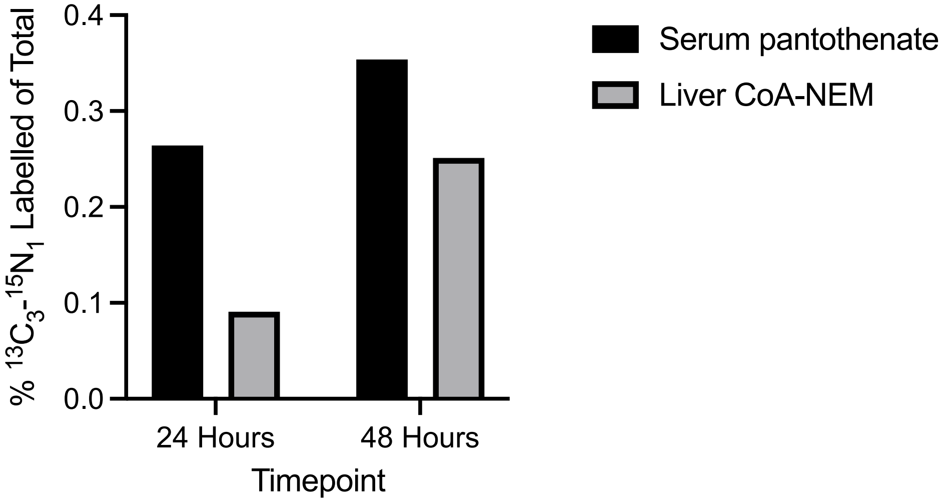

Supplement: Supplement 1 [file media-1.pdf]
